# Abnormal Cerebral Blood Flow and Functional Connectivity Strength in Subjects With White Matter Hyperintensities

**DOI:** 10.3389/fneur.2021.752762

**Published:** 2021-10-20

**Authors:** Hao Huang, Kun Zhao, Wenzhen Zhu, Hui Li, Wenhao Zhu

**Affiliations:** ^1^Department of Neurology, Tongji Hospital, Tongji Medical College, Huazhong University of Science and Technology, Wuhan, China; ^2^School of Biological Science & Medical Engineering, Beijing Advanced Innovation Center for Biomedical Engineering, Beihang University, Beijing, China; ^3^Department of Radiology, Tongji Hospital, Tongji Medical College, Huazhong University of Science and Technology, Wuhan, China

**Keywords:** white matter hyperintensities, arterial spin labeling, cerebral blood flow, functional magnetic resonance imaging, functional connectivity strength, neurovascular coupling

## Abstract

White matter hyperintensities (WMHs) are common neuroimaging findings in the aging population and are associated with various clinical symptoms, especially cognitive impairment. Abnormal global cerebral blood flow (CBF) and specific functional connections have been reported in subjects with higher WMH loads. Nevertheless, the comprehensive functional mechanisms underlying WMH are yet to be established. In this study, by combining resting-state functional magnetic resonance imaging and arterial spin labeling, we investigated the neurovascular dysfunction in subjects with WMH in CBF, functional connectivity strength (FCS), and CBF–FCS coupling. The whole-brain alterations of all these measures were explored among non-dementia subjects with different WMH loads using a fine-grained Human Brainnetome Atlas. In addition, exploratory mediation analyses were conducted to further determine the relationships between these neuroimaging indicators, WMH load, and cognition. The results showed that subjects with higher WMH loads displayed decreased CBF and FCS mainly in regions involving the cognitive- and emotional-related brain networks, including the default mode network, salience network, and central executive network. Notably, subjects with higher WMH loads also showed an abnormal regional CBF–FCS coupling in several regions of the thalamus, posterior cingulate cortex, and parahippocampal gyrus involving the default mode network. Furthermore, regional CBF in the right inferior temporal gyrus and right dorsal caudate may mediate the relationship between WMH load and cognition in WMH subjects. These findings indicated characteristic changes in cerebral blood supply, brain activity, and neurovascular coupling in regions involving specific brain networks with the development of WMH, providing further information on pathophysiology underpinnings of the WMH and related cognitive impairment.

## Introduction

White matter hyperintensities (WMHs), also known as leukoaraiosis or white matter (WM) lesions, are common neuroimaging findings in the aging population, which are typically described as hyperintensities on fluid-attenuated inversion recovery (FLAIR) magnetic resonance images (MRIs) (1, 2). Considered as established markers of cerebral small vessel disease (CSVD), WMHs are demonstrated to be associated with mood disorders, gait disturbance, and, particularly, cognitive impairment in various domains (1–3). Neuroimaging studies could directly investigate the structural and functional abnormalities in subjects with WMH *in vivo* and have provided important information on understanding the pathophysiological mechanisms underlying WMH (4–7). In the past decades, convergent evidence has identified the disruption of WM microstructural integrity and structural networks with the development of WMH and the related cognitive decline (6, 8, 9). However, compared with structural abnormalities, functional changes in subjects with WMH were much less reported *in vivo*, and a comprehensive picture of functional mechanisms underlying WMH is still far from clarified.

Previous studies at the cellular and the tissue level suggested that neurovascular dysfunction may be involved in the increase of WMH burden (10–13). Hence, hemodynamic indicators in neuroimaging studies, such as cerebral blood flow (CBF) and blood-oxygen-level-dependent (BOLD) functional magnetic resonance imaging (fMRI), may provide new perspectives for further elucidating the potential functional underpinnings of WMH (14). CBF is an important indicator that can reflect brain functional metabolism in respect of glucose utilization, oxygen consumption, and aerobic glycolysis (15). Using invasive imaging techniques of single-photon emission computed tomography, Xeon-computed tomography, or positron emission tomography, previous studies have indicated that subjects with higher WMH loads showed a decreased global CBF reduction in the whole brain, WM, and gray matter (GM) (16–20). Furthermore, a few recent studies have attempted to investigate CBF changes in WMH subjects using arterial spin labeling (ASL) MRI, which is a non-invasive technique without contrast and revealed a relationship between WMH load and CBF in WM, especially in the areas of WMH and normal-appearing WM around WMH (19–23). Nevertheless, these studies mainly focused on the global CBF changes of the whole brain or large brain areas in subjects with WMH, yet the characteristic alteration map of regional CBF in subjects with different WMH loads has not been evaluated. Resting-state BOLD fMRI is another promising neuroimaging tool for evaluating the aberrant functional architecture underlying the increase of WMH burden, which reflects the neuronal activity and intrinsic functional interactions and integrations among anatomically separated brain regions (24–27). Based on resting-state fMRI, previous studies have revealed aberrant functional connectivity (FC) in specific regions involving the default mode network (DMN) in subjects with higher WMH loads, which was associated with cognitive decline (25, 26, 28). As a data-driven, newly developed measure for FC calculation, the functional connectivity strength (FCS) represents the connectivity of each voxel with all other voxels in the brain (29–31). Hence, different from the seed-based FC analysis and independent component analysis approach used in previous studies (25, 26, 32), FCS analysis may be used to delineate overall functional alterations of each brain region underlying the increase of WMH burden.

Brain regions with higher neuronal activity usually tend to have higher perfusion and energy supply, indicating a neurovascular coupling that plays a critical role in maintaining normal brain function (33). Combined ASL and fMRI, previous studies have demonstrated a striking correlation between resting-state FCS and CBF in the normal brain (30, 34), further supporting the hypothesis of neurovascular coupling. Moreover, abnormal global and characteristic regional CBF–FCS coupling has been identified in several neuropsychiatric diseases, including schizophrenia, Wilson's disease, primary open-angle glaucoma, and generalized anxiety disorder (34–37). However, the relationship between CBF and FCS in subjects with different WMH loads remains unknown, which may provide complementary evidence for clarifying potential functional mechanisms in WMH.

Here, we hypothesized that the changes of not only CBF and FCS but also CBF–FCS coupling could contribute to the increase of WMH burden and the related cognitive impairment. To test this hypothesis, resting-state ASL and fMRI data were acquired to compute CBF, FCS, and correlation between CBF and FCS from 86 non-dementia subjects with WMH. Furthermore, to obtain the precise alteration information of CBF, FCS, and CBF–FCS coupling in both the whole GM and each brain sub-region, we investigated the global and local alteration patterns of these neuroimaging measures among subjects with different grades of WMH using a fine-grained Human Brainnetome Atlas (38). In addition, we performed exploratory correlation and mediation analyses to evaluate the relationships between these neuroimaging indicators, WMH load, and cognition.

## Materials and Methods

### Subjects

Eighty-six non-dementia subjects with WMH on MRI scans were recruited from the outpatient service of the Department of Neurology, Tongji Hospital, Wuhan, China. The inclusion criteria for the participants were as follows: (1) 50–80 years old, right-handedness; (2) mild to severe WMH; (3) >5 years of education; (4) no dementia based on the criteria of the Diagnostic and Statistical Manual of Mental Disorders, Fourth Edition; (5) no MRI contraindication. The exclusion criteria were as follows: (1) cerebral infarcts with the diameter >15 mm or cerebral hemorrhage; (2) WMH mimics (e.g., leukodystrophy and multiple sclerosis); (3) severe large vessel diseases, such as carotid artery stenosis (>50%); (4) systematic diseases (e.g., cancer, connective tissue diseases); (5) a history of epilepsy, degenerative diseases, such as Alzheimer's disease and Parkinson's disease, or psychiatric diseases; (6) could not fulfill the MRI scan or the neuropsychological tests. The severity of WMH was visually rated on FLAIR images according to the Fazekas rating scale by two experienced neurologists (W.H.Z. and H.H.) blinded to the clinical information. The final score was the sum of the deep WMH score (0—absent, 1—punctuate foci, 2—beginning confluence of foci, 3—large confluent areas) and the periventricular WMH score (0—absent, 1—caps or pencil lining, 2—smooth halo, 3—irregular periventricular hyperintensities extending into deep WM) (39, 40). The correlation coefficient of the results from the two observers and the inter-rater coefficient was higher than 0.9. When the two observers failed to reach an agreement on the WMH score, another experienced neurologist (S.B.X., with 20 years of experience) would evaluate the Fazekas score of the case independently. A definite evaluation would be made if two of the three observers agreed; otherwise, they would discuss the case to make the final decision. Furthermore, according to the different WMH loads, the participants were divided into three groups: the mild WMH group (the total Fazekas scores of 1 or 2, *n* = 32), the moderate WMH group (the total Fazekas scores of 3 or 4, *n* = 24), and the severe WMH group (the total Fazekas scores of 5 or 6, *n* = 30). The Ethics Committee of Tongji Hospital, Tongji Medical College, Huazhong University of Science and Technology, approved the study (ID: TJ-C20131216). Written informed consent was obtained from each participant.

### Neuropsychological Tests

All participants received a series of neuropsychological tests to examine the performance of various cognitive domains, including (1) global cognition: the Mini-Mental State Examination; (2) processing speed: the Trail Making Test Part A; (3) executive function: Trail Making Test Part B; (4) episodic memory: the Auditory Verbal Learning Test long-delayed recall (20 min); (5) and language: the Verbal Fluency Test (41). For comparisons of performance across each cognitive domain, raw scores for these neuropsychological tests were transformed to *z*-scores based on the means and standard deviations across all the WMH subjects included in the present study and were then presented as the cognitive performance of each domain (42).

### Data Acquisition

MRIs were acquired on a 3.0-T MR scanner (Discovery MR750, GE Healthcare, Milwaukee, WI, USA) using a 32-channel head array coil. T1-weighted images were collected using a BRAVO sequence with a repetition time (TR) of 8.16 ms, echo time (TE) of 3.18 ms, inversion time (TI) of 50 ms, flip angle (FA) of 12°, a matrix of 256 × 256, a field of view (FOV) of 256 × 256 mm, a slice thickness of 1 mm, and 188 slices. FLAIR images were acquired using a TR/TE of 8,000/160 ms, a TI of 2,100 ms, an FA of 111°, a 512 × 512 matrix, a 240 × 240-mm FOV, a slice thickness of 5.0 mm, and a slice gap of 1.5 mm. Resting-state fMRI data were obtained by an axial gradient echo-planar imaging sequence with a TR/TE of 2,000/35 ms, an FA of 90°, a 64 × 64 matrix, a 220 × 220-mm FOV, a slice thickness of 3.0 mm, a slice gap of 1.0 mm, and an acquisition time of 7 min. ASL MRI data were collected using a pseudo-continuous ASL technique, with a TR/TE of 5,086/15 ms, a TI of 2,025 ms, an FA of 111°, a 128 × 128 matrix, a 240 × 240-mm FOV, a slice thickness of 4.0 mm, and 50 slices. All the subjects were asked to relax, move as little as possible, and keep their eyes closed during the scan. Foam pads were used to minimize head movement, and earplugs were used to attenuate scanner noise.

### Cerebral Blood Flow Analysis

The ASL data were pre-processed using the CAT12 toolbox (Computational Anatomy Toolbox; http://www.neuro.uni-jena.de/cat) implemented in SPM12 (Statistical Parametric Mapping, Institute of Neurology, London, UK). First, the ASL images were calculated by subtraction of the label images and control images based on the single-compartment model; after that, the images were then converted to the CBF images combined with the proton-density-weighted reference images (34, 43). Second, the skulled structural three-dimensional T1-weighted images were registered to the standard Montreal Neurological Institute (MNI) space to obtain the deformation parameters. Furthermore, the proton-density-weighted reference images were registered to the subject's T1-weighted images and written to the standard MNI space using before obtained deformation parameters, then applied to the CBF images and resliced to a 3 × 3 × 3-mm cubic voxel.

### Functional Magnetic Resonance Imaging Data Pre-processing

The fMRI data were pre-processed by the Brainnetome fMRI toolkit (BRANT, http://brant.brainnetome.org) (44). The first 10 volumes were removed because of the magnetization equilibration effect, and the remaining images were corrected by the slice timing procedure to solve the acquisition time delay between different slices. The images were then realigned to the first volume to correct for head motion. Of note, we restricted the further analysis in participants with no more than 3° angular rotation on any axis or 3-mm translation, and no subjects were excluded according to the criteria. Next, the images were normalized to the MNI space and resampled to 3 × 3 × 3-mm voxels. Furthermore, a linear regression analysis was conducted to remove nuisance variables, including the linear shift, the mean signals from CSF and WM, and the head motion at the *x*-, *y*-, and *z*-axes. Finally, to eliminate the effect of low-frequency drifts and high-frequency noise, the temporal band-pass filter (0.01–0.08 Hz) was applied.

### Functional Connectivity Strength Analysis

The Pearson's correlation coefficients were calculated between all pairs of the whole GM voxels, and the functional connectivity (FC) matrix was obtained from each participant. To reduce the influence of background noise, we restricted the analysis to positive correlations above a threshold of 0.2, according to previous studies that reported specific alterations of resting-state FCS in several neuropsychological diseases (34, 36, 37, 45–47). If the value of an FC was lower than 0.2, it would be set to zero. The FCS of a given voxel was calculated as the average of the FC between the voxel and all the remaining voxels (30, 45).

### Cerebral Blood Flow–Functional Connectivity Strength Coupling Analysis

To quantitatively explore the relationship between CBF and FCS in subjects with WMH, we conducted correlation analyses across voxels and subjects, according to the protocols proposed by Liang et al. (30). Before the CBF–FCS coupling analysis, both ASL and BOLD images were smoothed by a 6-mm full width half maximum Gaussian kernel, and for each participant, CBF and FCS values were transferred into *z*-scores by subtracting mean and dividing by the standard deviation of global values within the GM as previous studies reported (30, 34). A distinctive CBF–FCS correlation coefficient was generated for each subject at the global GM level. Similarly, regional CBF–FCS coupling for each subject was calculated by averaging the CBF–FCS correlation coefficients of all voxels containing in a certain brain region. To delineate the whole-brain CBF–FCS decoupling maps with the increase of WMH burden, both the global and regional CBF–FCS correlations were further compared among the three groups in the study.

### Statistical Analyses

#### Between-Group Differences in Demographic and Clinical Data

The differences in demographic and clinical data, including medical history and neuropsychological scores among the three groups, were evaluated by one-way analysis of variance (ANOVA), the Kruskal–Wallis test, and the χ^2^ test using SPSS 20.0 (IBM Corp., Armonk, NY, USA). To explore the potential effects of head motion, we also compared framewise displacement (48), a representative metric for head motion among the three groups. The relationship between the WMH scale and the z-score of each cognitive test was also determined by partial correlation analysis, controlling for age, sex, and years of education.

Atlas-Based Comparison in Cerebral Blood Flow, Functional Connectivity Strength, and Cerebral Blood Flow–Functional Connectivity Strength Coupling

We performed one-way ANOVA and *post-hoc* analysis to identify differences in the FCS, CBF, and correlation coefficient between CBF and FCS among the three WMH groups at the whole GM level (*p* < 0.01, permutation test for 1,000 times), with age and sex as confounding variables. To further characterize the regional changes of these neuroimaging measures of each subject, the human Brainnetome Atlas consists of 246 subregions in the cerebrum (210 cortical and 36 subcortical regions) (http://atlas.brainnetome.org) and has been widely used in previous neuroimaging studies that were used (38). The cerebellum regions were not included in the analysis in the study. One-way ANOVA and *post-hoc* analysis were also performed to evaluate the characteristic regional changes in all these neuroimaging measures among the three WMH groups, with age and sex as covariates (*p* < 0.01, permutation test for 1,000 times).

#### Exploratory Mediation Analyses

We performed Pearson's correlation analyses to evaluate the relationships between the CBF, FCS, and coefficient of CBF–FCS correlation in different brain regions and the z-scores of each cognitive domain in all subjects with WMH, controlling for age, sex, and years of education (*p* < 0.05). Furthermore, to determine whether the relationship between WMH load and cognitive decline in WMH subjects can be explained by the changes of hemodynamic measures revealed in our study, exploratory mediation analyses were implemented using the PROCESS macro v3.1 in SPSS (49). In all mediation models, the altered neuroimaging measures identified in the between-group comparisons, including CBF, FCS, and correlation coefficient between CBF and FCS, were separately entered as the mediators, whereas the WMH scale of each individual was entered into the models as the predictor and the z-score of each cognitive domain as the outcome, age, sex, and years of education as confounding variables. Mediation analyses were evaluated using a bootstrap method (*n* = 5,000), and significant indirect effects were defined by a 95% confidence interval, not including zero.

#### Control Analysis

To verify the stability of our main results, we repeated the between-group comparisons by adding hypertension and diabetes as covariates to investigate the potential confusion of the two risk factors. Furthermore, 16 matched healthy controls (HCs) from the in-house database with no visible WMH on FLAIR images were additionally included in the analysis to validate the alteration patterns of CBF, FCS, and CBF–FCS coupling in subjects with higher WMH loads (the details can be found in the Supplementary Material).

## Results

### Demographic, Structural Magnetic Resonance Imaging, and Neuropsychological Data Analysis

Demographic, neuropsychological, and structural MRI characteristics of the participants are summarized in Table 1, Supplementary Table 1. There were no significant differences in age, gender, years of education, global gray matter volume, and framewise displacement among the three groups. The subjects in the moderate WMH and the severe WMH groups showed more proportion of hypertension and diabetes history than the subjects in the mild WMH group, whereas no significant differences were observed in these metrics between the moderate WMH group and the severe WMH group. Subjects in the severe WMH group exhibited worse performance in global cognitive function, executive function, and episodic memory compared with the other two groups and exhibited worse performance in processing speed compared with the moderate WMH group. No difference in language was observed among the three groups.

**Table 1:** Demographic and clinical features of subjects.

	**Mild WMH (*n* = 32)**	**Moderate WMH (*n* = 24)**	**Severe WMH (*n* = 30)**	***p*-value**
**Demographic data**
Age (years)	63.69 ± 5.47	64.33 ± 6.65	66.43 ± 5.07	0.126^a^
Gender (male/female)	16/16	13/11	18/12	0.731^b^
Education (years)	10.78 ± 3.13	11.08 ± 3.69	9.10 ± 3.89	0.078^a^
Hypertension (yes/no)	6/26	17/7^d^	21/9^e^	<0.001^b^
Diabetes (yes/no)	3/29	4/20	8/22	0.199^b^
Smoking history (yes/no)	5/27	7/17	10/20	0.250^b^
Drinking history (yes/no)	8/24	3/21	3/27	0.234^b^
**MRI features**
Gray matter volume (ml)	614.96 ± 38.54	615.65 ± 31.16	626.83 ± 57.33	0.517^c^
Framewise displacement	0.18 ± 0.16	0.18 ± 0.10	0.17 ± 0.08	0.957^c^
**Cognitive performance**
Global cognitive function	0.28 ± 0.69	0.32 ± 0.79	−0.56 ± 1.19^e^^,^^f^	<0.001^c^
Processing speed	0.18 ± 0.88	0.26 ± 0.89	−0.39 ± 1.11 ^f^	0.026^c^
Executive function	0.33 ± 0.76	0.20 ± 0.95	−0.52 ± 1.08^e^^,^^f^	0.001^c^
Episodic memory	0.34 ± 0.88	0.12 ± 1.00	−0.46 ± 0.98^e^^,^^f^	0.005^c^
Language	0.25 ± 0.85	0.09 ± 1.12	−0.33 ± 0.99	0.061^c^

a *Kruskal–Wallis test*.

b *χ^2^ test*.

c *One-way analysis of variance*.

d *Significant difference between mild WMH and moderate WMH groups*.

e *Significant difference between mild WMH and severe WMH groups*.

f *Significant difference between moderate WMH and severe WMH groups*.

*WMH, white matter hyperintensities*.

### Spatial Distribution of Cerebral Blood Flow and Functional Connectivity Strength

**Figure 1:**
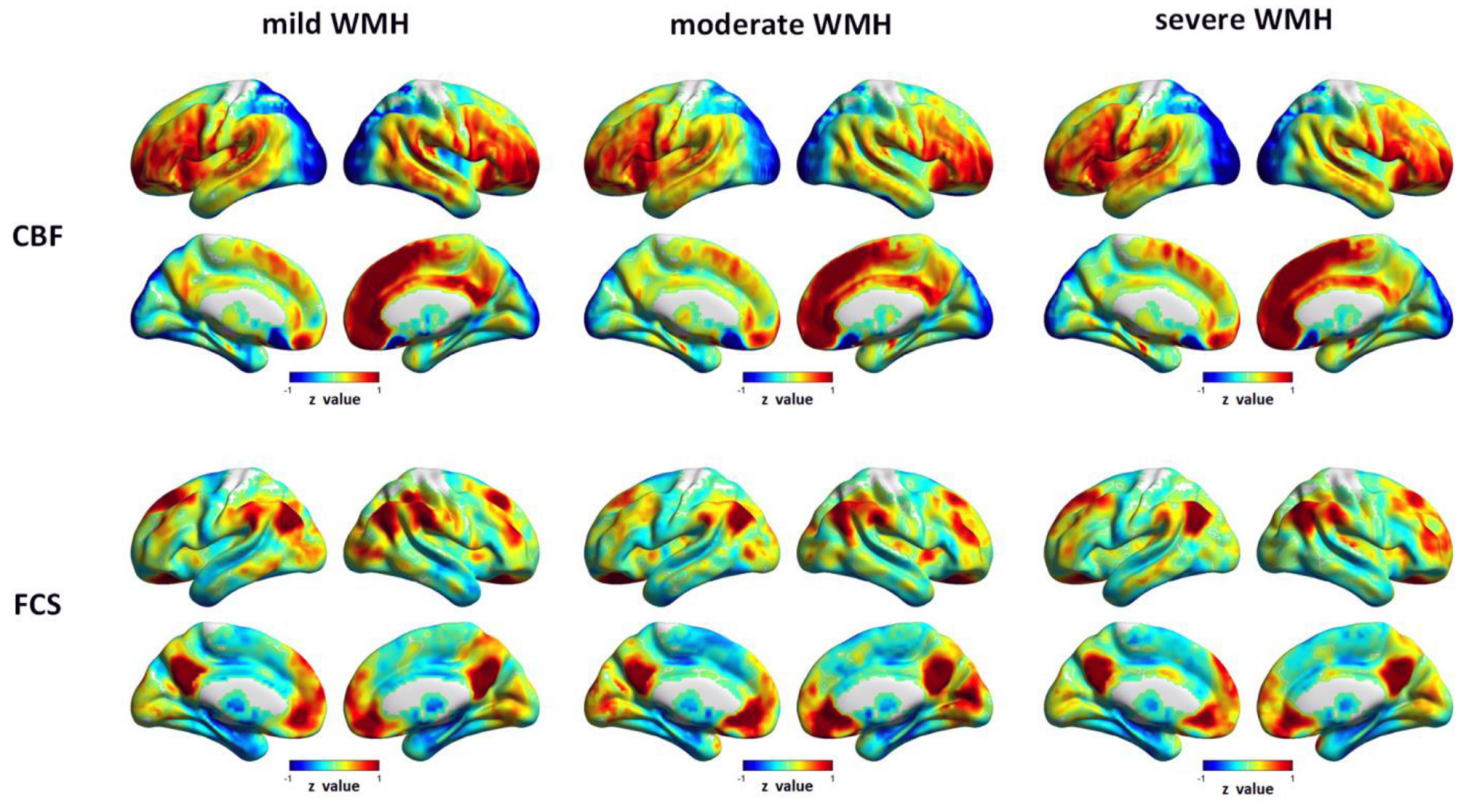
Spatial distribution maps CBF and FCS in subjects with different WMH grades. CBF and FCS maps are normalized to *z*-values and averaged among subjects within each group. CBF, cerebral blood flow; FCS, functional connectivity strength; WMH, white matter hyperintensities.

Visually, the distributions of CBF and FCS were similar among the three WMH groups (Figure 1). All of the three groups exhibited higher values of CBF in the posterior cingulate gyrus (PCC)/precuneus (PCu), anterior cingulate cortex (ACC), bilateral medial frontal cortex, lateral parietal cortex, lateral pre-frontal cortex (LPFC), lateral temporal cortex, insula, and several thalamus regions, most of which were core brain regions of the DMN and salience network (SN). Brain regions with higher FCS values were primarily located in the DMN regions, LPFC, insula, and visual cortex, showing a similar distribution pattern with CBF.

### Cerebral Blood Flow and Functional Connectivity Strength Changes Among Subjects With Different White Matter Hyperintensity Loads

From the mild WMH group to the severe WMH group, both global CBF and global FCS showed a decreasing trend in whole GM. Particularly, subjects in the severe WMH group displayed a prominent decrease of both global CBF and global FCS in whole GM compared with the mild WMH group (Supplementary Table 2).

**Figure 2:**
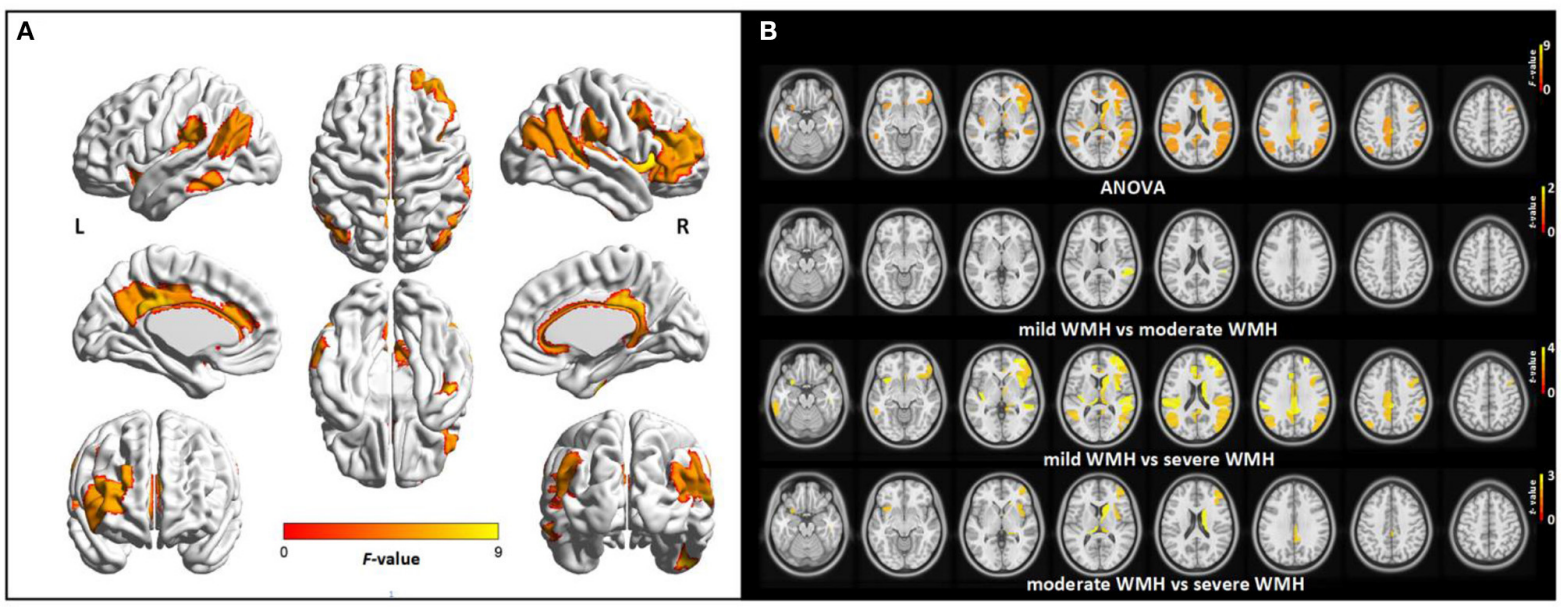
Group differences in regional CBF among subjects with different WMH loads controlling for age and sex. **(A)** Surface statistical maps displaying *F*-scores of ANOVA for altered CBF among three groups (*p* < 0.01, with permutation test for 1,000 times). **(B)** Axial statistical maps displaying *F*-scores of ANOVA and *t-*scores of *post-hoc* analysis between every two groups. In statistical maps of ANOVA, warmer colors indicate significant differences in local CBF among three groups. In statistical maps of *post-hoc* analysis between every two groups, warm colors represent regional CBF decrease in moderate WMH group compared with mild WMH group, or in severe WMH group compared with mild WMH group, or in WMH3 group compared with moderate WMH group, respectively. L, left; R, right.

The identified brain regions showing altered CBF among the three groups mainly included the widespread cortical regions covering the PCC, ACC, bilateral inferior parietal lobule (IPL), dorsolateral pre-frontal cortex (DLPFC), frontal operculum, insula, and several regions of the temporal cortex, and subcortical regions of the bilateral thalamus and right dorsal caudate. Compared with the mild WMH group, subjects in the severe WMH group showed significantly decreased CBF in all these regions, whereas patients in the severe WMH group also showed lower CBF in the bilateral insula, thalamus, and a few regions of the frontal and temporal cortex, PCC, and caudate compared with the moderate WMH group. Significant altered CBF between the moderate WMH group and mild WMH group was observed in only one sub-region of the temporal cortex (Figure 2, Supplementary Table 3).

**Figure 3:**
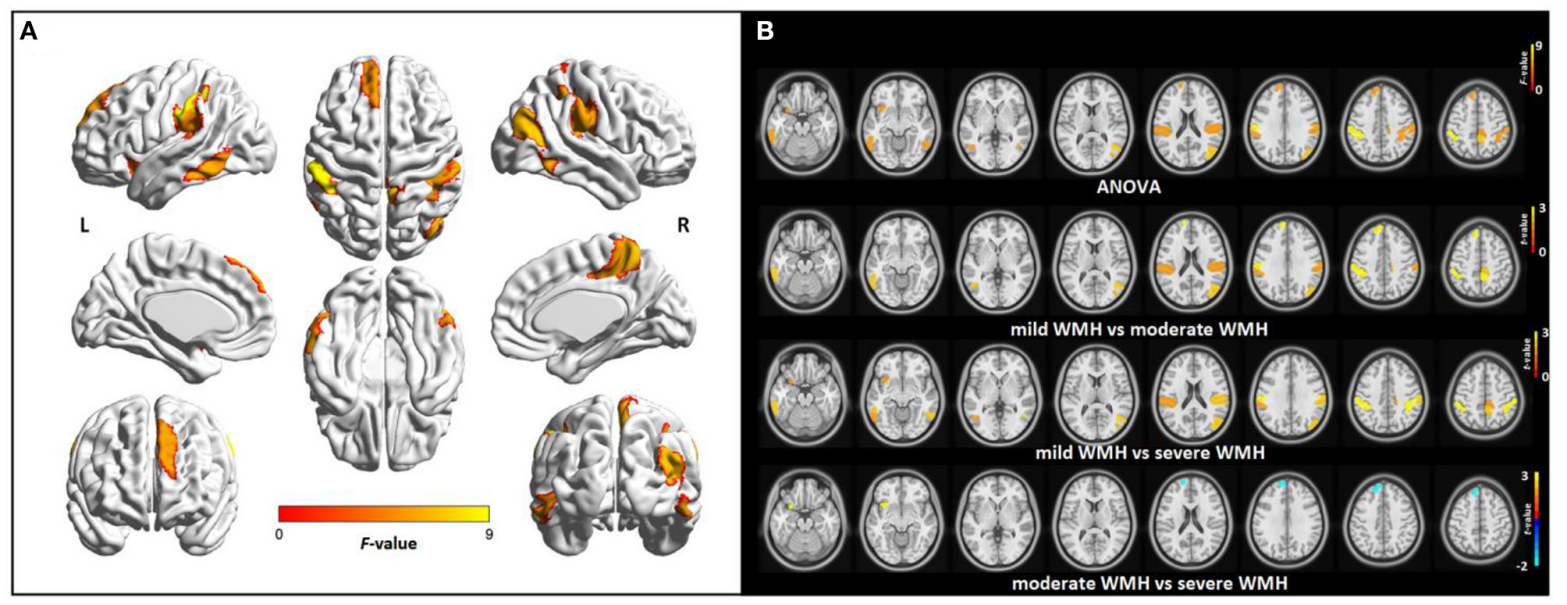
Group differences in regional FCS among subjects with different WMH loads controlling for age and sex. **(A)** Surface statistical maps displaying *F*-scores of ANOVA for altered FCS among three groups (*p* < 0.01, with permutation test for 1,000 times). **(B)** Axial statistical maps displaying *F*-scores of ANOVA and *t*-scores of *post-hoc* analysis between every two groups. In statistical maps of ANOVA, warmer colors indicate significant differences in regional FCS among three groups. In statistical maps of *post-hoc* analysis between every two groups, warm colors represent regional FCS decrease in moderate WMH group compared with mild WMH group, or in severe WMH group compared with mild WMH group, or in WMH3 group compared with moderate WMH group, respectively, and cold colors represent regional FCS increase in severe WMH group compared with moderate WMH group. No significant increased FCS was found in severe WMH group relative to mild WMH group, or moderate WMH group when compared with mild WMH group. L, left; R, right.

The FCS alteration map among the three WMH groups is shown in Figure 3. Relative to the mild WMH group, the patients in the severe WMH group exhibited lower FCS primarily in the bilateral IPL, inferior temporal gyrus (ITG), left anterior insula, left DLPFC, and PCun. Different from the CBF change pattern, lower FCS values in the moderate WMH group could also be observed in several regions of the IPL, ITG, and left DLPFC as compared with the mild WMH group. Furthermore, the subjects in the severe WMH group showed significantly decreased FCS in the left anterior insula and showed significantly increased FCS in the left DLPFC, compared with the moderate WMH group. In addition, the full overlap of the CBF and FCS changes was located in several regions of the IPL and anterior insula (Supplementary Table 4).

#### Global and Regional Cerebral Blood Flow–Functional Connectivity Strength Coupling Changes Among Subjects With Different White Matter Hyperintensity Loads

**Figure 4:**
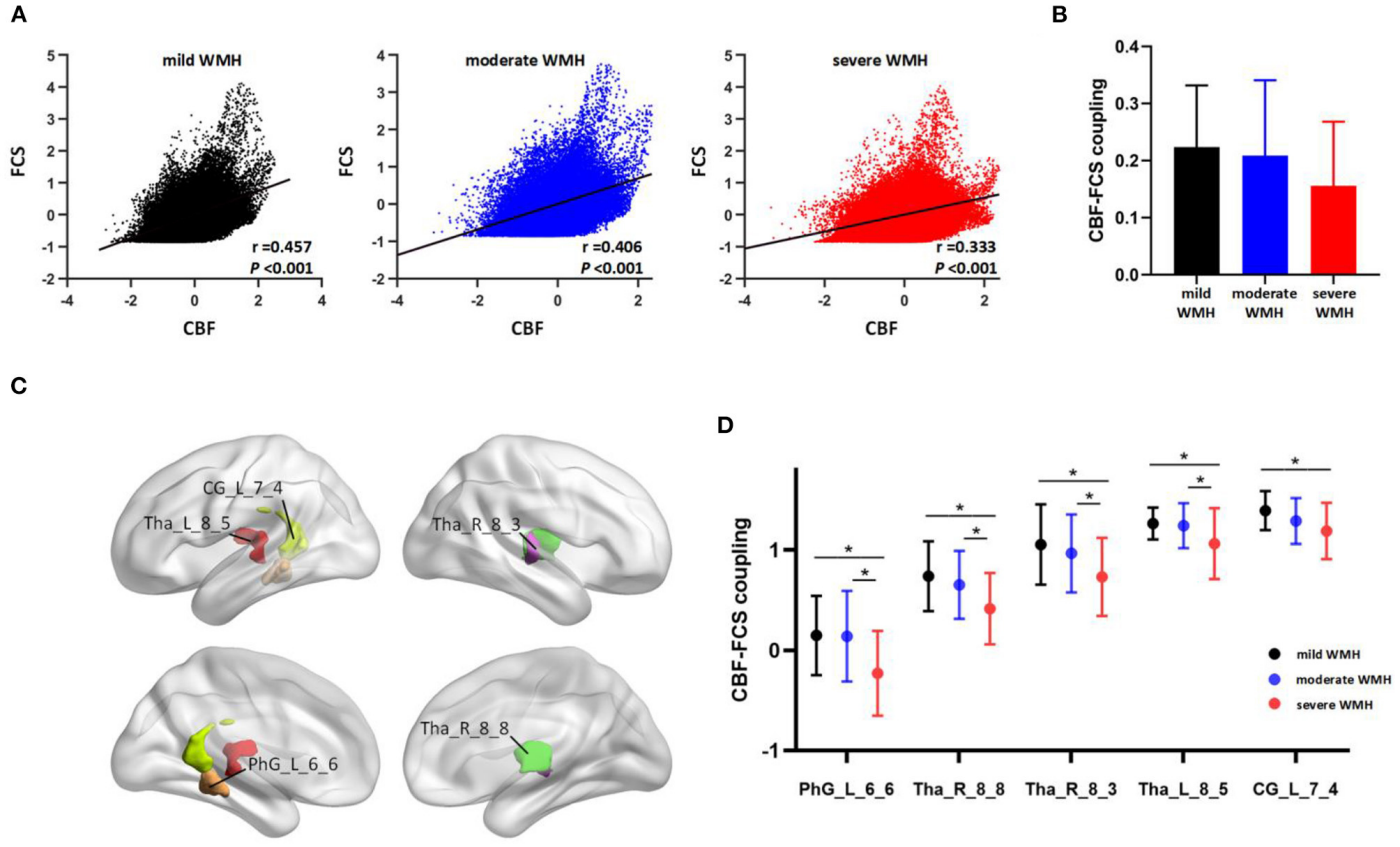
Global and regional CBF–FCS coupling changes among subjects with different WMH loads. **(A)** Whole-brain CBF–FCS coupling alterations across voxels in samples of subjects with different WMH loads. **(B)** Between-group comparisons exhibited a decreasing trend in global CBF–FCS coupling from mild WMH group to severe WMH group (mild WMH group: *r* = 0.225 ± 0.108; moderate WMH group: *r* = 0.209 ± 0.132; severe WMH group: *r* = 0.156 ± 0.112), whereas no significant differences were identified in global CBF–FCS coupling among three WMH groups (*F* = 1.645, *p* = 0.199). **(C,D)** Altered regional CBF–FCS coupling among three WMH groups. Compared with mild WMH and moderate WMH group, severe WMH group showed significant aberrant CBF–FCS coupling in bilateral thalamus, ACC, and left parahippocampal gyrus. Data were shown as adjusted correlation coefficients between CBF and FCS controlling for age and sex. Error bars represent the SD. *p < 0.05. ACC, anterior cingulate cortex; PhG, parahippocampal gyrus; Tha, thalamus; WMH, white matter hyperintensities. L, left; R, right. Detailed information on brain regions is available at http://atlas.brainnetome.org/.

In all of the three WMH groups, CBF was significantly correlated with FCS across voxels of the whole GM (Figure 4A). However, the global CBF–FCS coupling showed a decreasing trend with the increase of WMH load, although no significant difference was identified in the correlation coefficient among the three groups (Figure 4B). The severe WMH group showed significantly reduced CBF–FCS coupling in several regions of the bilateral thalamus compared with the other two WMH groups and showed significantly reduced CBF–FCS coupling in the PCC compared with the mild WMH group. In addition, compared to the other two groups, the severe WMH group showed a significantly altered CBF–FCS correlation pattern that CBF was negatively associated with FCS in a sub-region of the left parahippocampal gyrus (Figures 4C,D, Supplementary Table 5).

#### Relationships Between Neuroimaging Measures, White Matter Hyperintensity Load, and Cognitive Performance

**Figure 5:**
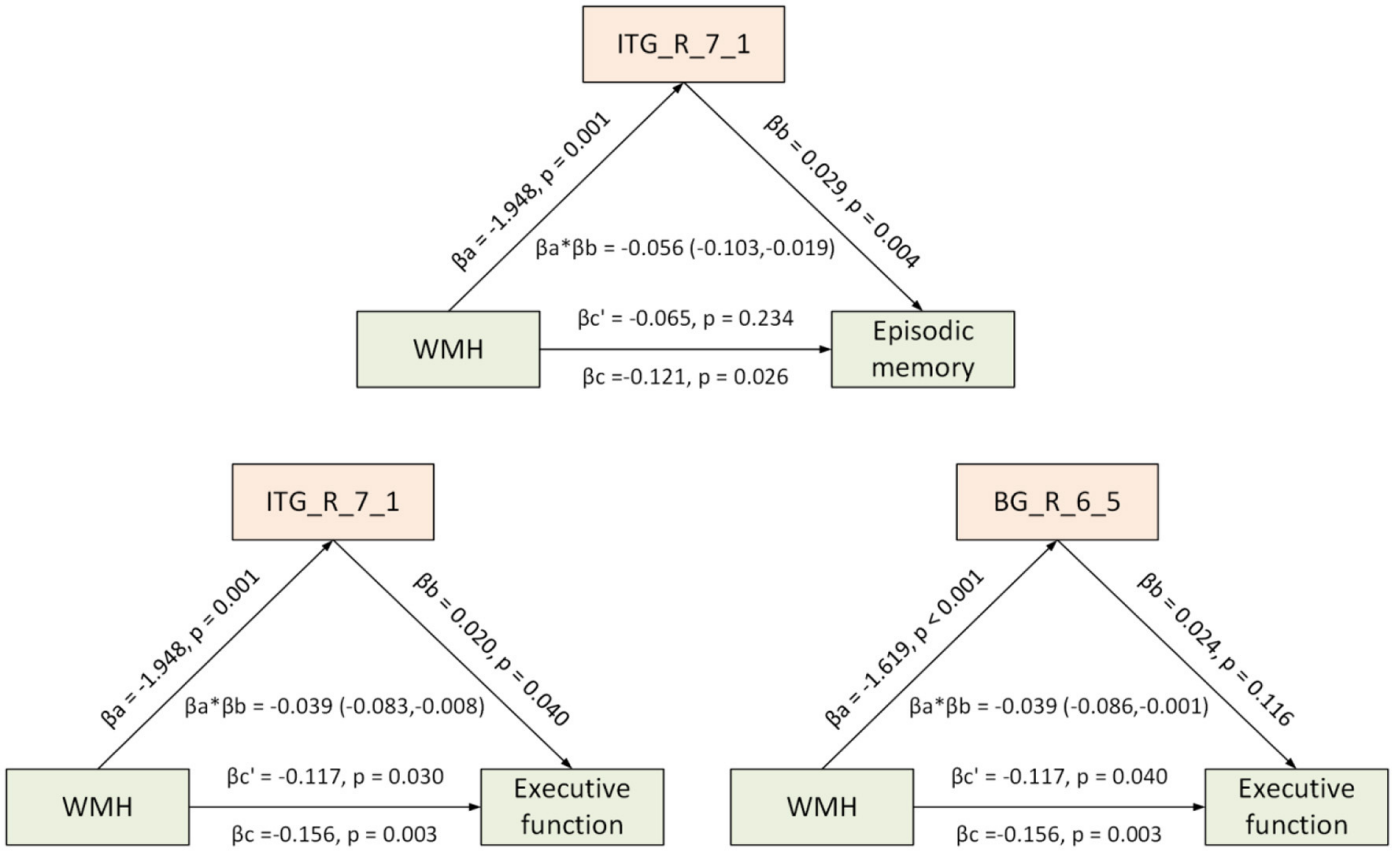
Mediation effect of regional CBF between WMH load and cognitive function. Diagrams show areas of CBF mediating relationship between WMH load (represented as Fazekas score) and cognitive performance (executive function or episodic memory), and mediation models of pathways from WMH load to CBF reduction in right ITG and right caudate to reduce cognitive performance. In each model, age, sex, and years of education were entered as covariates. βa and βb represent coefficients of relationships between WMH load and regional CBF, and associations between region CBF and cognition (when both WMH load and CBF were both entered into model as predicting variables). Total effect (βc) = Direct effect (βc') + Mediation effect (βa*βb). Mediating role of regional CBF reduction on association between WMH and cognition is defined when 95% confidence interval did not conclude 0 for 5,000 bootstrapping iterations. BG, basal ganglia; CBF, cerebral blood flow; FCS, functional connectivity strength; ITG, inferior temporal gyrus; WMH, white matter hyperintensities. L, left; R, right. Detailed information on brain regions is available at http://atlas.brainnetome.org/.

In all WMH subjects, there were significant associations between WMH Fazekas score and global cognitive function (*r* = −0.299, *p* = 0.006), executive function (*r* = −0.323, *p* = 0.003), episodic memory (*r* = −0.244, *p* = 0.026), and language (*r* = −0.217, *p* = 0.049), and no significant association was identified between Fazekas score and processing speed (*r* = −0.188, *p* = 0.089). CBF in several regions of frontal, temporal, parietal, insular, cingulate cortices, and caudate was found to be positively associated with cognitive decline in various domains, especially processing speed and executive function (Supplementary Table 6). Meanwhile, the FCS–cognition relationship was observed only between regional FCS in a sub-region of IPL and executive function, and the correlation coefficient between CBF and FCS in a sub-region of the thalamus was significantly associated with global cognitive performance (Supplementary Table 6). Because no significant correlation was identified between WMH load and processing speed, here mediation models were established only for the cognitive performance of other domains. Significant mediation effects of WMH on the cognitive decline were observed via regional CBF in a sub-region of ITG (for episodic memory and executive function) and right dorsal caudate (for executive function) (Figure 5, Supplementary Table 7).

#### Control Results

For all of the three neuroimaging measures (CBF, FCS, and CBF–FCS coupling), significant correlations between the *F*-scores or *t*-scores in the main results with and without controlling for hypertension and diabetes were observed (Supplementary Figures 1–3), indicating that other risk factors, such as hypertension and diabetes, are unlikely to provide a convincing explanation of alterations in the hemodynamic measures underlying the increase of WMH burden revealed in this study. The patterns of differences in all the three measures between the HC group and the moderate/severe WMH group were spatially consistent with alteration patterns in these measures between the mild WMH group and the other two WMH groups (Supplementary Figures 4–6, Supplementary Table 8), emphasizing the robustness of our main results that revealed characteristic alteration patterns of these measures in subjects with higher WMH loads.

## Discussion

To the best of our knowledge, this is the first study to depict the whole-brain CBF, FCS, and CBF–FCS coupling alteration maps among subjects with different WMH loads by combining ASL and BOLD fMRI techniques. Subjects with higher WMH loads showed decreased CBF and FCS in subcortical and cortical regions involving several important large-scale brain networks, especially the DMN, SN, and CEN. Notably, patients with severe WMH load showed abnormal regional CBF–FCS coupling in several regions of the thalamus, PCC, and parahippocampal gyrus. Furthermore, exploratory mediation analyses identified the crucial role of specific CBF reduction underlying cognitive impairment in subjects with WMH. Altogether, these findings provide further information on dysfunctional mechanisms underlying WMH and related cognitive impairment from the comprehensive perspective of blood supply, brain activity, and neurovascular decoupling.

In the present study, we found a significant trend of both CBF and FCS reduction with the increase of WMH load. As neuroimaging measures reflecting distinct brain functional aspects of metabolic demand and neuronal activity, respectively, both CBF and FCS can be affected by structural damages (50, 51). Prior works have demonstrated that the disruption of WM fibers underlay the increase of WMH burden and was associated with various clinical symptoms related to WMH, including cognitive impairment, gait disturbance, and depression (6, 52, 53). Severe and extensive WM disconnections can result in the degeneration and hypometabolism of the GM connected to the impaired WM fibers, which may be an important explanation for the recorded CBF and FCS reduction in subjects with more severe WMH (54, 55). Another possible mechanism for CBF and FCS changes is the abnormalities in GM attributed to CSVD directly. Considered to be the primary neuroimaging manifestations of CSVD, WMHs are usually accompanied by other damages in GM, including cortical microinfarcts, cerebral microbleeds, and GM atrophy, which may lead to the lower blood supply required by GM tissues as well as the decreased neuronal activity (56). In addition, aberrant inflammatory factors, which are considered to be involved in the pathophysiological mechanisms of WMH, would also contribute to the abnormal modulation of CBF and FCS in subjects with higher WMH loads (13).

Of note, our results showed a characteristic regional CBF decrease in cortical functional hub regions, which exhibited higher values of CBF in all the three groups, mainly covering the core component of the DMN (PCC/PCu, bilateral IPL, and lateral temporal cortex) (57, 58), SN (ACC, bilateral frontal operculum, and insula) (58, 59), and CEN (bilateral DLPFC and posterior parietal cortex) (58), as well as subcortical nuclei, which also serve as integrative hubs for brain networks (bilateral thalamus and right dorsal caudate) (60, 61) in subjects with more WMH. Similarly, significantly decreased FCS in subjects with moderate and severe WMH were detected mainly in core nodes of the DMN (PCu, bilateral IPL, and lateral temporal cortex) (57, 58), ECN (bilateral posterior parietal cortex and right DLPFC) (58), and SN (left anterior insula) (58, 59). Particularly, a full overlap of the CBF and FCS changes in subjects with higher WMH loads was identified in several of these regions, including IPL and anterior insula. The DMN, CEN, and SN are the most robust intrinsic large-scale networks that account for a wide range of cognitive and emotional processing (58). Consistent with previous studies focused on the structural and functional abnormalities in WMH (25, 26, 28, 62), the characteristic alteration patterns of both CBF and FCS among subjects with different WMH loads emphasized that the dysfunction of these intrinsic brain networks may play a crucial role in the pathophysiology of WMH. It is worth noting that there were also some differences in the alteration patterns of CBF and FCS among subjects with diverse WMH loads. Altered CBF was mainly identified between the severe WMH group and the other two groups, whereas FCS alterations were mainly detected in the comparisons between the mild WMH group and the two groups with higher WMH loads. Because only subjects in the severe WMH group showed significantly impaired cognitive performance, one could speculate that the disconnections of functional networks may appear earlier than CBF reduction with the increase of WMH burden, and extensive WM disconnections, as well as microvasculature damage associated with neuroinflammation, may further generate hypoperfusion in cognitive-related networks, resulting in cognitive impairment in subjects with WMH. Another possible explanation is that referred to as the “degree centrality” of weighted networks, FCS could not fully reflect the whole alteration picture of dissociable intrinsic brain networks independently (31). For example, FCS may be normal when significantly increased FCs and decreased FCs coexist involving a certain voxel or region. Hence, more information of aberrant intra- and inter-network connectivity architecture, which may attribute to the further increase of WMH burden and the subsequent cognitive decline, would not be completely revealed by FCS alterations. In addition, in the severe WMH group, the values of FCS in the left DLPFC were significantly higher than those in the moderate WMH group but were still significantly lower than those in the mild WMH group, which might be explained as a compensatory mechanism to the structural impairments of the DLPFC in the patients with severe WMH; on the other hand, DLPFC plays important roles not only in a wide range of cognitive domains but also in modulating emotional processing (59, 63–65). Thus, in line with previous studies focused on major depression and schizophrenia (63, 66), this result may be related to emotion dysregulation in patients with severe WMH as reported.

Another strength of our study is that we explored the alterations of both the global and regional CBF–FCS coupling among the subjects with different WMH loads. The whole-brain CBF–FCS coupling showed a decreasing trend from the mild WMH group to the severe WMH group, and importantly, subjects with severe WMH load showed significantly altered regional CBF–FCS correlations in several core nodes of the DMN, including the thalamus, PCC, and parahippocampal gyrus (57, 67). The altered associations between CBF and FCS within the DMN in the subjects with more WMH, which were first discovered by our study from the novel aspect of neurovascular decoupling, further emphasized that the selective functional abnormalities in the DMN may play a key role in the increase of WMH burden in addition to our results of CBF and FCS alterations. The neurovascular coupling depends on various components and their interaction of neurovascular unit, including neurons, astrocytes, and vessels (68). One possible explanation for neurovascular decoupling in subjects with a high WMH load is the prominent astrocyte dysfunction, as previous studies reported, which may impede the interaction between neuronal activity and vascular response (13, 33). Second, the vascular factors, especially endothelial cell dysfunction together with a series of concomitant inflammatory processes, which have been considered as another important pathological basis for WMH, may lead to the dysregulation of the CBF and neuronal activity (10–13, 69). Finally, GM atrophy in WMH subjects due to the denervation associated with WM disconnections and the microvasculature damages in GM, which has been demonstrated in various structural neuroimaging studies (5, 70–72), may also contribute to the reduced neurovascular coupling in subjects with higher WMH loads. Because no difference in global gray matter volume was found among the three groups in our study, further investigations with a larger sample size are needed to verify this hypothesis.

Previous studies have reported that resting-state functional connectivity within the DMN was associated with cognitive decline in subjects with WMH (26, 28). Consisting with and further extending their findings, our results showed significant correlations between cognitive performance and all the functional neuroimaging measures investigated in our study (CBF, FCS, and CBF–FCS coupling) in several cortical and subcortical hub regions involving cognitive networks in subjects with WMH. In particular, the results of mediation analyses indicated that CBF in the right ITG and right dorsal caudate might mediate the relationship between WMH load and cognition. Thought to be a constituent of the DMN, the ITG is well-known to be involved in various high-cognitive functions, including visual perception, language comprehensions, and memory (73–75). The right dorsal caudate has also been demonstrated to constitute a core circuit supporting the integration of diverse cognitive networks and is involved in the process of executive function (60, 76). Thus, we infer that cortical and subcortical hub regions involving essential cognitive and emotional networks would be more vulnerable to the neurovascular damage caused by WMH, resulting in a series of emotional and cognitive impairment, and specific regional CBF may serve as a potential neuroimaging marker for detecting an early cognitive decline in the future. However, no mediation effect of either regional FCS or CBF–FCS coupling between WMH and cognitive function was identified, which may partly be attributed to relatively simple neuropsychological scales to reflect cognitive performance in each domain and the selection of threshold in the FCS calculation. Because this is the first time to investigate the alterations of FCS and CBF–FCS coupling in the subjects with WMH, these findings should be further evaluated in the future. In addition, it is worth noting that many factors could contribute to cognitive decline in older adults, and the relationship between WMH load and cognitive impairment revealed by our study as well as previous works would only be considered as a correlation but not causality. Hence, our results may only provide a possible explanation to the association between WMH burden and cognitive impairment using mathematical models, and the detailed mechanisms of how hemodynamic changes regulate the WMH progression and lead to cognitive impairment should be further investigated.

The current study has several limitations that should be addressed. First, as CBF at rest varies greatly with time, it may be insufficient to provide precise information on regional cerebral perfusion and the real demand of blood supply by tissues (69, 77). Other alternative measures, for instance, cerebrovascular reactivity, may be applied to explore further the adequacy of tissue-level CBF in the subjects with different WMH loads (78, 79). Second, this is an exploratory study with relatively small sample size, and few regions can survive with a strict multiple comparison correction in this study. Here, we added to introduce a permutation test (for 1,000 times, *p* < 0.01) for the comparison of each measure among the three groups to increase the reliability of the results. Third, the control analysis showed that whether compared with the mild WMH group or the HC group, regional CBF, FCS, and CBF–FCS coupling in subjects with higher WMH loads exhibited very similar alteration patterns, highlighting the important role of dysfunction in brain regions involving large-scale networks underlying the increase of WMH load. Nevertheless, given the very small sample size in the additional HC group, there were still limitations in these findings. Fourth, in line with several previous studies (30, 34–37, 47), the procedure of partial volume correction was not implemented in this study. Also, in the between-group CBF analysis, we did not conduct a mean-centering of CBF at the individual level to compare the differences in absolute regional CBF among subjects with different WMH loads. Finally, we did not collect the information on emotional and gait disturbance common in subjects with a high WMH load. Large-scale longitudinal studies with more detailed clinical information will be warranted to determine further the exact role of neurovascular dysfunction in the increase of WMH burden and related clinical symptoms.

In conclusion, we investigated dysfunctional architectures in WMH subjects in the alterations in CBF, FCS, and coupling between CBF and FCS among the subjects with diverse WMH loads. Specifically, we found decreased regional CBF, FCS, and disrupted CBF–FCS coupling in hub regions involving cognitive- and emotional-related networks in subjects with higher WMH loads. These findings provide novel insights into the pathophysiology underpinnings of WMH and related cognitive impairment.

## Data Availability Statement

The original contributions presented in the study are included in the article/Supplementary Material, further inquiries can be directed to the corresponding author/s.

## Ethics Statement

The studies involving human participants were reviewed and approved by the Ethics Committee of Tongji Hospital, Tongji Medical College, Huazhong University of Science and Technology. The patients/participants provided their written informed consent to participate in this study.

## Author Contributions

WenhZ conceived and designed the experiments. WenhZ, HH, and HL recruited the subjects and collected the clinical and neuropsychological information of the subjects. WenzZ was responsible for MRI data acquisition. HH, KZ, and WenhZ performed data analysis. WenhZ and HH wrote the manuscript. All authors contributed to the article and approved the submitted version.

## Funding

This work was supported by the National Natural Science Foundation of China (81801146, 81871438, and 81771341) and the Beijing Natural Science Funds for Distinguished Young Scholars (JQ200036).

## Conflict of Interest

The authors declare that the research was conducted in the absence of any commercial or financial relationships that could be construed as a potential conflict of interest.

## Publisher's Note

All claims expressed in this article are solely those of the authors and do not necessarily represent those of their affiliated organizations, or those of the publisher, the editors and the reviewers. Any product that may be evaluated in this article, or claim that may be made by its manufacturer, is not guaranteed or endorsed by the publisher.

## Abbreviations

ACC: anterior cingulate cortex
ANOVA: one-way analysis of variance
ASL: arterial spin labeling
BOLD: blood-oxygen-level-dependent
CBF: cerebral blood flow
CEN: central executive network
CSVD: cerebral small vessel disease
DLPFC: dorsolateral prefrontal cortex
DMN: default mode network
FC: functional connectivity
FCS: functional connectivity strength
fMRI: functional magnetic resonance imaging
GM: gray matter
LPFC: lateral prefrontal cortex
IPL: inferior parietal lobule
ITG: inferior temporal gyrus
MRI: magnetic resonance imaging
PCC: post**-**cingulate cortex
SN: salience network
TMT: Trail Making Test
WM: white matter
WMH: white matter hyperintensities.
