# Supplementary material for: Abnormal Cerebral Blood Flow and Functional Connectivity Strength in Subjects With White Matter Hyperintensities
Source: Front Neurol. 2021 Oct 20;12:752762. doi: 10.3389/fneur.2021.752762 (PMC8564178; doi:10.3389/fneur.2021.752762)
Supplement: Supplementary file 1 [file Data_Sheet_1.pdf]

Supplementary Material for

Abnormal Cerebral Blood Flow and Functional Connectivity Strength in Subjects with White Matter Hyperintensities

Supplementary Methods

To validate the alteration patterns of CBF, FCS and CBF-FCS coupling in subjects with higher WMH loads, 16 age-, sex-, and education- matched subjects without any WMH or cognitive impairment from the in-house database were additionally included as healthy controls (HCs) in the control analysis. Written informed consent has been obtained from each participant. The HCs should meet the following criteria: 1) 50-80 years old, right-handedness; 2) no visible WMH (Fazekas scale of 0); 3) > 5 years of education; 4) no subjective cognitive complaints or objective cognitive impairment; 5) no MRI contraindication. The HCs should also not meet the exclusion criteria for subjects with WMH included in this study and undergo the same protocols of neuropsychological assessment and MRI scans. In addition, we restricted the further analysis in participants with no excessive head motion (no more than 3° angular rotation on any axis or 3 mm translation), and no HCs were excluded according to the criteria.

The same quality control criteria and preprocessing pipeline described in the main text were applied to the HCs. In consideration of the very small sample size in the HC group, for each neuroimaging measure, here we just conducted correlation analyses between *t*-scores of the HC group vs the moderate/severe WMH group and *t*-scores of the mild WMH group vs moderate/severe WMH group to verify the alteration patterns of these measures in subjects with higher WMH loads. Pearson's correlation coefficient of *t*-scores of *t*-test between groups (with age and sex controlled) was calculated at the regional level.

Supplementary Table 1. Raw neuropsychological data of the three WMH groups.

	Mild WMH (n = 32)	Moderate WMH (n = 24)	Severe WMH (n = 30)	Overall p Value
MMSE	28.78 ± 1.48	28.88 ± 1.68	27.00 ± 2.53[c,d]	< 0.001[a]
VFT	23.72 ± 5.06	22.58 ± 6.93	20.27 ± 5.83	0.061[b]
TMT-A	70.74 ± 37.91	67.27 ± 38.52	93.86 ± 47.00[d]	0.048[a]
TMT B	132.88 ± 54.06	140.21 ± 67.82	193.06 ± 79.00[c,d]	0.001[b]
AVLT long delay recall	7.03 ± 2.44	6.46 ± 2.64	4.77 ± 2.69[c]	0.005[b]

The data are presented as the mean ± SD.

[a] Kruskal-Wallis test.

[b] One-way analysis of variance.

[c] Significant difference between the mild WMH and severe WMH groups.

[d] Significant difference between the moderate WMH and severe WMH groups.

AVLT, Auditory Verbal Learning Test; Mini-Mental State Examination TMT, Trail Making Test; VFT, Verbal Fluency Test.

Supplementary Table 2. Differences in global CBF and FCS among the three groups.

	Mean CBF			ANOVA	
	**Mild WMH**	**Moderate WMH**	**Severe WMH**	***F***	***p***
CBF (ml/100 g/min)	57.51±10.94	53.98±10.31	49.54±9.37 [a]	3.8722	0.0247
FCS	0.0067±0.0033	0.0054±0.0025	0.0048±0.0020 [a]	5.2421	0.0072

Absolute values of CBF and FCS in whole grey matter in the three groups are shown as the mean ± SD. Age and sex were entered as covariates in the analysis.

[a] Significant difference between the mild WMH and severe WMH groups.

F, *F*-value of ANOVA. CBF, cerebral blood flow; FCS, functional connectivity strength; WMH, white matter hyperintensities.

Supplementary Table 3. Differences in regional CBF among the three groups.

Brain Area	Mean CBF			ANOVA		Mild WMH vs moderate WMH		Mild WMH vs severe WMH		Moderate WMH vs severe WMH	
	Mild WMH	Moderate WMH	Severe WMH	*F*	*p*	*t*	*p*	*t*	*p*	*t*	*p*
BG_R_6_5	49.719±7.539	47.539±4.896	43.895±4.908	7.3358	0.0012	1.2339	0.2226	3.5792	0.0007	2.7141	0.0090
CG_L_7_1	78.534±13.153	72.214±13.783	65.790±13.474	6.9594	0.0016	1.7433	0.0870	3.7678	0.0004	1.7233	0.0908
CG_L_7_3	60.637±10.244	57.146±11.050	51.947±9.032	5.8020	0.0044	1.2200	0.2277	3.5337	0.0008	1.9034	0.0625
CG_L_7_6	59.676±10.567	55.875±10.923	50.992±10.684	5.1035	0.0081	1.3130	0.1947	3.2162	0.0021	1.6522	0.1045
CG_R_7_1	89.311±15.879	83.357±15.616	74.927±14.482	6.8527	0.0018	1.3983	0.1678	3.7189	0.0004	2.0531	0.0451
CG_R_7_2	78.420±11.303	74.790±10.569	69.932±8.346	5.4333	0.0061	1.2224	0.2269	3.3452	0.0014	1.8882	0.0646
CG_R_7_4	77.150±14.410	72.781±9.758	67.264±11.353	5.0886	0.0082	1.2798	0.2061	2.9871	0.0041	1.8869	0.0648
IFG_R_6_2	67.224±13.710	62.674±12.339	56.731±10.793	5.5830	0.0053	1.2818	0.2054	3.3333	0.0015	1.8866	0.0648
IFG_R_6_5	65.123±11.587	60.614±10.105	56.765±8.374	5.2716	0.0070	1.5209	0.1341	3.2364	0.0020	1.5307	0.1319
INS_L_6_1	65.301±8.607	61.505±10.972	57.182±9.522	5.5071	0.0057	1.4517	0.1524	3.5261	0.0008	1.5493	0.1274
INS_L_6_2	77.954±7.773	77.641±12.867	70.649±9.233	5.1207	0.0080	0.1130	0.9105	3.3780	0.0013	2.3234	0.0241
INS_R_6_3	67.193±10.456	62.484±9.827	57.151±9.029	8.1287	0.0006	1.7108	0.0929	4.0355	0.0002	2.0741	0.0430
INS_R_6_6	57.640±8.733	55.127±8.237	50.073±7.731	6.6714	0.0021	1.0917	0.2798	3.6034	0.0006	2.3190	0.0244
IPL_L_6_5	55.828±12.252	52.218±13.243	45.659±12.432	5.1400	0.0079	1.0540	0.2966	3.2430	0.0019	1.8717	0.0669
IPL_L_6_6	64.057±9.454	59.843±12.231	54.973±10.245	5.7320	0.0047	1.4552	0.1514	3.6312	0.0006	1.5924	0.1174
IPL_R_6_1	59.884±13.963	53.776±13.374	49.003±12.941	5.1009	0.0081	1.6491	0.1049	3.1766	0.0024	1.3270	0.1903
IPL_R_6_5	50.670±13.760	43.680±13.194	40.047±12.115	5.3054	0.0068	1.9144	0.0609	3.2175	0.0021	1.0524	0.2975
IPL_R_6_6	65.804±13.420	60.441±12.287	55.990±10.005	5.1981	0.0075	1.5337	0.1310	3.2472	0.0019	1.4678	0.1482
ITG_R_7_1	51.246±10.382	47.661±9.545	42.285±7.140	7.5205	0.0010	1.3232	0.1913	3.9342	0.0002	2.3679	0.0216
MFG_R_7_2	62.884±13.750	57.379±10.754	53.389±9.728	5.1955	0.0075	1.6230	0.1104	3.1198	0.0028	1.4290	0.1590
MFG_R_7_3	64.933±12.413	59.186±13.100	54.238±11.714	5.8043	0.0044	1.6746	0.0998	3.4836	0.0009	1.4632	0.1494
MFG_R_7_4	77.601±14.440	73.198±13.448	66.291±11.114	5.8431	0.0042	1.1624	0.2502	3.4391	0.0011	2.0669	0.0437

OrG_R_6_6	67.199±11.199	62.311±9.474	58.839±9.649	5.2489	0.0071	1.7240	0.0904	3.1393	0.0026	1.3244	0.1912
PCun_L_4_4	81.908±14.505	75.612±14.507	69.391±16.159	5.3195	0.0067	1.6075	0.1138	3.2137	0.0021	1.4702	0.1475
pSTS_L_2_2	53.198±11.058	48.058±12.966	43.324±12.956	5.0085	0.0088	1.5984	0.1158	3.2341	0.0020	1.3336	0.1881
pSTS_R_2_1	47.305±11.979	41.045±11.349	37.709±10.276	5.8290	0.0043	1.9788	0.0529	3.3749	0.0013	1.1318	0.2629
pSTS_R_2_2	43.680±12.592	36.798±11.539	32.550±10.783	7.1550	0.0014	2.0968	0.0407	3.7265	0.0004	1.3944	0.1691
STG_R_6_2	61.283±11.679	56.574±11.653	51.949±9.991	5.4665	0.0059	1.4946	0.1408	3.3710	0.0013	1.5698	0.1225
Tha_L_8_7	58.175±9.867	55.954±9.908	48.639±11.022	7.1081	0.0014	0.8320	0.4090	3.5937	0.0007	2.5333	0.0144
Tha_R_8_1	61.334±12.803	57.803±9.898	51.956±11.321	5.1796	0.0076	1.1219	0.2669	3.0474	0.0034	1.9927	0.0516
Tha_R_8_6	67.443±11.545	65.096±10.490	58.996±9.080	5.3041	0.0068	0.7825	0.4374	3.1878	0.0023	2.2894	0.0261
Tha_R_8_7	75.014±11.516	71.516±9.208	63.993±9.920	9.0265	0.0003	1.2229	0.2267	4.0252	0.0002	2.8580	0.0061

CBF in the three groups is shown as the mean ± SD (p <0.01, permutation test for 1,000 times), controlling for age and sex. F, F-value of ANOVA; t, t-score of the post hoc analysis. CBF, cerebral blood flow; FCS, functional connectivity strength; WMH, white matter hyperintensities.

BG, basal ganglia; CG, cingulate gyrus; IFG, inferior frontal gyrus; INS, insular gyrus; IPL, inferior parietal lobule; ITG, inferior temporal gyrus; MFG, middle frontal gyrus; OrG, orbital gyrus; Pcun, Precuneus; pSTS, Posterior superior temporal sulcus; STG, superior temporal gyrus; Tha, thalamus; L, left; R, right. Detailed information on the brain regions is available at http://atlas.brainnetome.org/.

Supplementary Table 4. Differences in regional FCS among the three groups.

Brain Area	Mean FCS			ANOVA		Mild WMH vs moderate WMH		Mild WMH vs severe WMH		Moderate WMH vs severe WMH	
	Mild WMH	Moderate WMH	Severe WMH	*F*	*p*	*t*	*p*	*t*	*p*	*t*	*p*
INS_L_6_2	-0.0125±0.0061	-0.0105±0.0084	-0.0156±0.0034	4.8833	0.0099	-1.0445	0.3009	2.4598	0.0168	3.0612	0.0035
IPL_L_6_3	0.0249±0.0057	0.0204±0.0041	0.0200±0.0043	9.6844	0.0002	3.2460	0.0020	3.8120	0.0003	0.4011	0.6900
IPL_L_6_6	0.0198±0.0075	0.0158±0.0040	0.0158±0.0046	4.9544	0.0093	2.3788	0.0209	2.5322	0.0140	0.0033	0.9974
IPL_R_6_1	0.0222±0.0100	0.0164±0.0045	0.0160±0.0056	6.7035	0.0020	2.6358	0.0109	2.9625	0.0044	0.2559	0.7990
IPL_R_6_3	0.0202±0.0056	0.0178±0.0055	0.0157±0.0045	5.8128	0.0043	1.5655	0.1233	3.4895	0.0009	1.5868	0.1186
IPL_R_6_6	0.0207±0.0082	0.0163±0.0047	0.0153±0.0056	5.8666	0.0041	2.3277	0.0237	2.9786	0.0042	0.6928	0.4915
ITG_L_7_6	0.0191±0.0084	0.0146±0.0040	0.0145±0.0037	5.9199	0.0040	2.4292	0.0185	2.7745	0.0074	0.1176	0.9069
ITG_R_7_5	0.0108±0.0066	0.0080±0.0036	0.0067±0.0027	5.9782	0.0038	1.9034	0.0623	3.1194	0.0028	1.4295	0.1588
PCL_R_2_1	0.0263±0.0067	0.0222±0.0025	0.0228±0.0042	5.6476	0.0050	2.7901	0.0073	2.3995	0.0195	-0.6050	0.5478
PCun_R_4_2	0.0168±0.0059	0.0128±0.0022	0.0133±0.0044	6.6769	0.0020	3.1631	0.0026	2.6267	0.0109	-0.5338	0.5957
SFG_L_7_3	0.0349±0.0082	0.0284±0.0040	0.0322±0.0081	5.5311	0.0056	3.5881	0.0007	1.2729	0.2080	-2.1289	0.0380

FCS in the three groups is shown as the mean ± SD (p<0.01, permutation test for 1,000 times), controlling for age and sex. F, F-value of ANOVA; t, t-score of the post hoc analysis. FCS, functional connectivity strength; WMH, white matter hyperintensities.

CG, cingulate gyrus; INS, insular gyrus; IPL, inferior parietal lobule; ITG, inferior temporal gyrus; PCL, paracentral lobule; PCun, Precuneus; STG, superior temporal gyrus; L, left; R, right. Detailed information on the brain regions is available at http://atlas.brainnetome.org/.

Supplementary Table 5. Differences in regional CBF and FCS coupling among the three groups.

Brain Area	Mean coefficient of CBF-FCS coupling			ANOVA		Mild WMH vs moderate WMH		Mild WMH vs severe WMH		Moderate WMH vs severe WMH	
	Mild WMH	Moderate WMH	Severe WMH	*F*	*p*	*t*	*p*	*t*	*p*	*t*	*p*
CG_L_7_4	1.394±0.195	1.289±0.229	1.190±0.281	5.7123	0.0047	1.8451	0.0705	3.3338	0.0015	1.3959	0.1687
PhG_L_6_6	0.148±0.396	0.141±0.451	-0.229±0.423	7.6993	0.0009	0.0680	0.9460	3.6279	0.0006	3.0958	0.0032
Tha_L_8_5	1.264±0.158	1.243±0.224	1.065±0.353	5.3419	0.0066	0.4090	0.6841	2.8998	0.0052	2.1511	0.0361
Tha_R_8_3	1.055±0.400	0.967±0.388	0.733±0.389	5.4671	0.0059	0.8229	0.4142	3.2112	0.0021	2.2033	0.0320
Tha_R_8_8	0.740±0.349	0.653±0.338	0.416±0.356	7.0457	0.0015	0.9288	0.3571	3.6199	0.0006	2.4908	0.0160

The correlation coefficient between regional CBF and FCS in the three groups is shown as the mean ± SD (p <0.01, permutation test for 1,000 times), controlling for age and sex. *F*, *F*-value of ANOVA; *t*, *t*-score of the post hoc analysis. CBF, cerebral blood flow; FCS, functional connectivity strength; WMH, white matter hyperintensities.

CG, cingulate gyrus; PhG, parahippocampal gyrus; Tha, thalamus; L, left; R, right. Detailed information on the brain regions is available at http://atlas.brainnetome.org/.

Supplementary Table 6. Regions in which significant correlations between hemodynamic neuroimaging measures and cognitive performance in subjects with WMH subjects were identified.

**Brain region**	**r**	***p***
Significant associations between regional CBF and global cognitive function in subjects with WMH		
ITG_R_7_1	0.238	0.030
Significant associations between regional CBF and processing speed in the subjects with WMH		
ITG_R_7_1	0.280	0.010
IFG_R_6_2	0.219	0.047
IFG_R_6_5	0.239	0.029
OrG_R_6_6	0.254	0.020
STG_R_6_2	0.249	0.023
INS_L_6_1	0.258	0.018
INS_R_6_6	0.228	0.038
BG_R_6_5	0.219	0.047
Significant associations between regional CBF and executive function in the subjects with WMH		
ITG_R_7_1	0.314	0.004
IFG_R_6_2	0.290	0.008
IFG_R_6_5	0.245	0.025
MFG_R_7_2	0.239	0.030
OrG_R_6_6	0.254	0.021
STG_R_6_2	0.301	0.006
IPL_R_6_6	0.229	0.037
INS_L_6_1	0.260	0.018
INS_R_6_6	0.266	0.015
CG_R_7_2	0.221	0.044
BG_R_6_5	0.290	0.008

Significant association between regional CBF and episodic memory in the subjects with WMH		
ITG_R_7_1	0.368	0.001
Significant association between regional FCS and executive function in subjects with WMH		
IPL_L_6_3	0.268	0.014
Significant associations between regional CBF-FCS coupling coefficient and global cognitive function in subjects with WMH		
Tha_L_8_5	0.221	0.045

BG, basal ganglia; CG, cingulate gyrus; IFG, inferior frontal gyrus; INS, insular gyrus; IPL, inferior parietal lobule; ITG, inferior temporal gyrus; MFG, middle frontal gyrus; OrG, orbital gyrus; STG, superior temporal gyrus; Tha, thalamus; L, left; R, right. Detailed information on the brain regions is available at http://atlas.brainnetome.org/.

Supplementary Table 7. Mediation effect of CBF on the relationship between WMH scale and cognition.

Mediators	Path a		Path b		Direct effect (path c')		Mediation effect (path a*b)	
	β	*p*	β	*p*	β	*p*	β	95%CI
Mediation effect on the association between WMH scale and executive function, with age, sex, education as covariates. Total effect: βc=-0.156 *p* =0.003.								
ITG_R_7_1	-1.948	0.001	0.020	0.040	-0.117	0.030	-0.039	(-0.083,-0.008)
BG_R_6_5	-1.619	<0.001	0.024	0.116	-0.117	0.040	-0.039	(-0.086,-0.001)
Mediation effect on the association between WMH scale and episodic memory, with age, sex, education as covariates. Total effect: βc=-0.121 *p* =0.026								
ITG_R_7_1	-1.948	0.001	0.029	0.004	-0.065	0.234	-0.056	(-0.103,-0.019)

The table shows the areas in which regional CBF mediate the relationship between WMH and cognition. Mediation analysis was used to assess the potential indirect relationship between WMH load (presented as Fazekas score) (X) and cognitive performance (Y) via mean CBF in regions identified by between-group analyses (M). In each model, age, sex, years of education were entered as covariates. Paths a and b indicate the association between WMH load and regional CBF, and the associations between regional CBF and cognition (when both WMH load and regional CBF were entered into the model as predictive variables), respectively. Path c represents the total effect of WMH load on cognition, and path c' shows the direct effect of WMH load on cognition after controlling for regional CBF as a mediating factor. The mediating role of regional CBF on the association between WMH load and cognition is defined by the 95% bootstrap CI for 5,000 bootstrapping iterations. Significant mediation effects were defined by a 95% CI entirely above or below 0. Total effect(βc) = Direct effect(βc') + Mediation effect(βa*βb).

β, standardized regression coefficient; CI, confidence interval; X, predictor variable; Y, outcome variable; M, mediator; BG, basal ganglia; ITG, inferior temporal gyrus; L, left; R, right. Detailed information on the brain regions is available at http://atlas.brainnetome.org/.

Supplementary Table 8. Demographic, neuropsychological and MRI features of the healthy controls.

**Demographic data**	
Age (years)	60.50 ± 7.64
Gender (male/female)	10/6
Education (years)	11.00 ± 3.79
Hypertension (yes/no)	1/15
Diabetes (yes/no)	0/16
Smoking history (yes/no)	5/11
Drinking history (yes/no)	5/11
**Neuropsychological data**	
MMSE	29.00 ± 1.15
VFT	24.00 ± 11.64
TMT-A	74.87 ± 28.97
TMT B	130.31 ± 58.13
AVLT long delay recall	7.88 ± 2.72
**MRI features**	
Gray matter volume (ml)	613.96 ± 40.56
Framewise displacement	0.12 ± 0.05

No significant differences in age, sex, years of education, grey matter volume and framewise displacement were identified among the HC group and the three WMH groups. There were also no significant differences in other demographic, neuropsychological and neuroimaging metrics between the HC group and the mild WMH group.

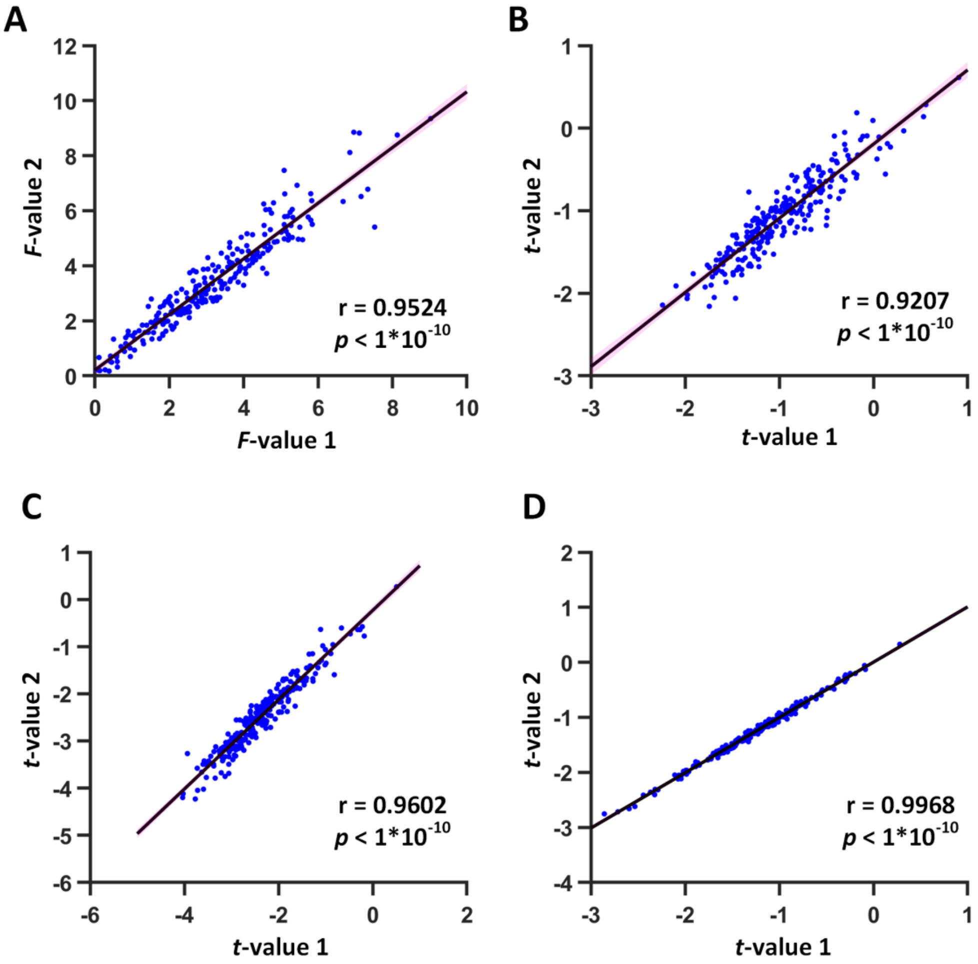

Supplementary Figure 1. Correlation analyses of the among-group differences in regional CBF with and without controlling for hypertension and diabetes. (A) The scatter plot of correlation analyses between the *F*-scores of ANOVA with (y-axis) and without (x-axis) controlling for hypertension and diabetes. (B, C, D) The scatter plots showing the correlation analyses between the *t*-scores of the moderate WMH group vs the mild WMH group (B), the *t*-scores of the severe WMH group vs the mild WMH group (C), and the *t*-scores of the severe WMH group vs the moderate WMH group (D), with (y-axis) and without (x-axis) controlling for hypertension and diabetes.

r, Pearson's correlation coefficient. CBF, cerebral blood flow.

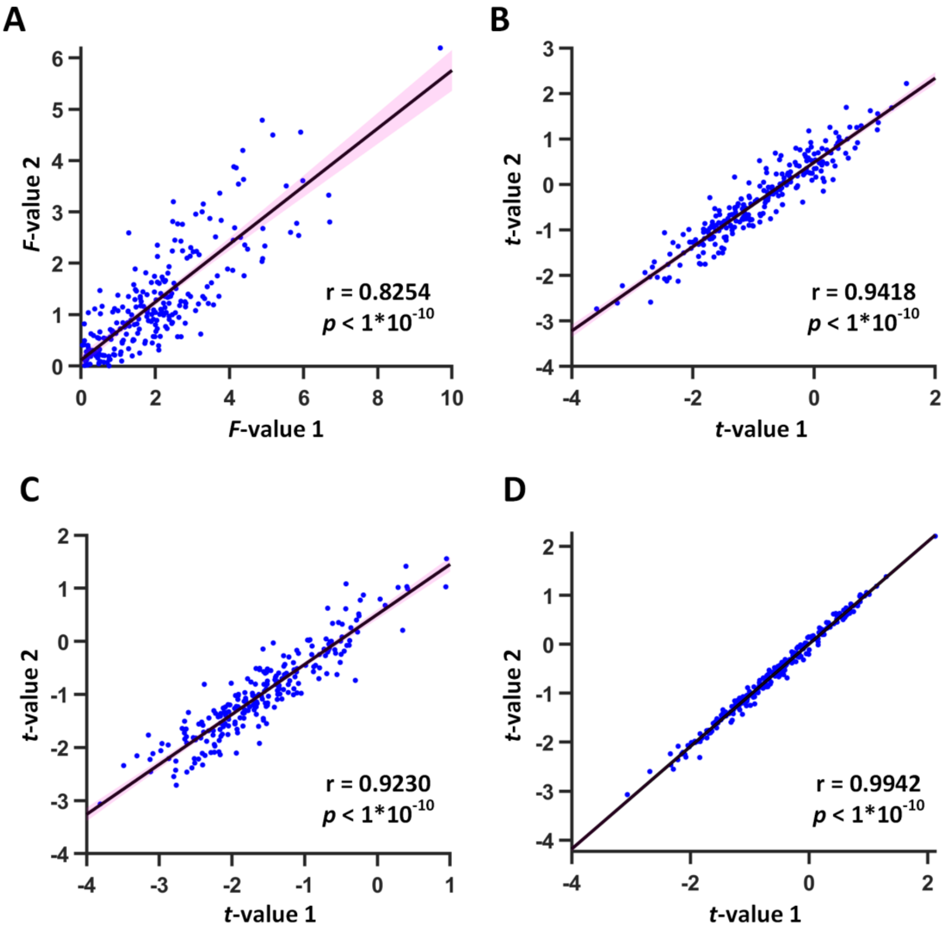

Supplementary Figure 2. Correlation analyses of the among-group differences in regional FCS with and without controlling for hypertension and diabetes. (A) The scatter plot of correlation analyses between the *F*-scores of ANOVA with (y-axis) and without (x-axis) controlling for hypertension and diabetes. (B, C, D) The scatter plots showing the correlation analyses between the *t*-scores of the moderate WMH group vs the mild WMH group (B), the *t*-scores of the severe WMH group vs the mild WMH group (C), and the *t*-scores of the severe WMH group vs the moderate WMH group (D), with (y-axis) and without (x-axis) controlling for hypertension and diabetes.

r, Pearson's correlation coefficient. FCS, functional connectivity strength.

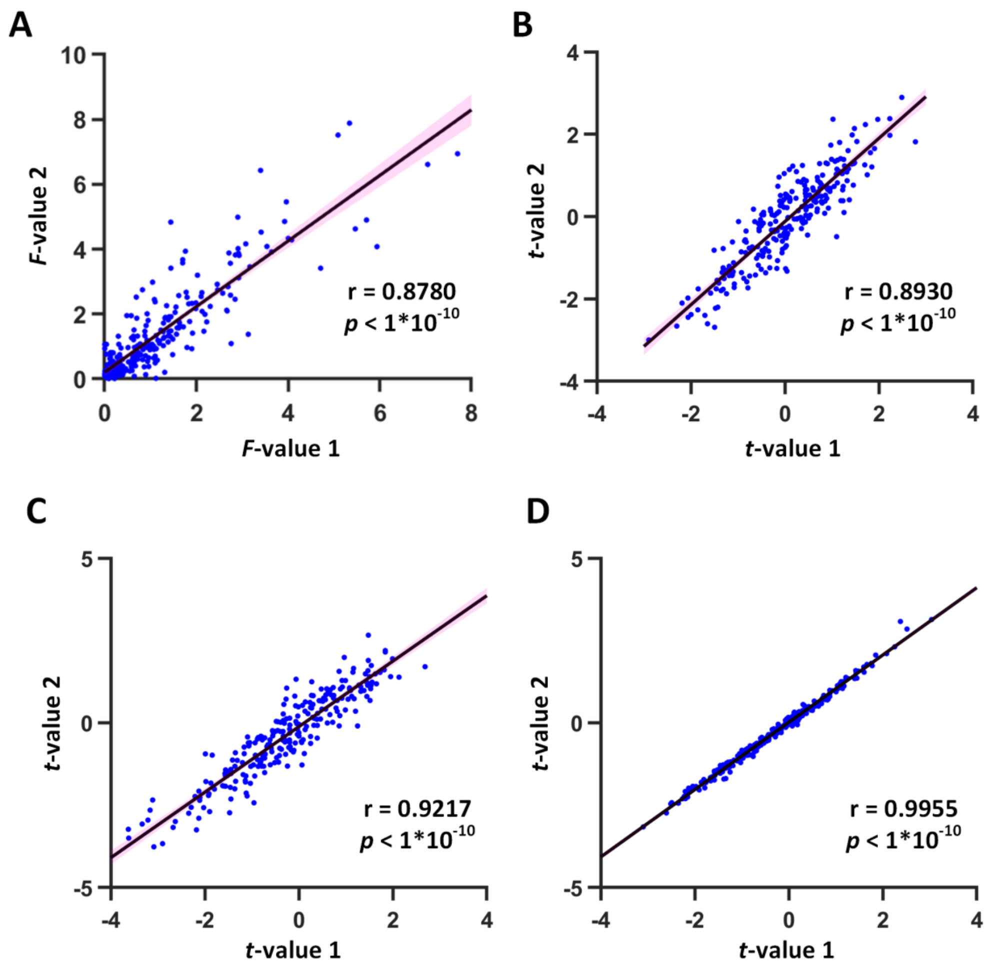

Supplementary Figure 3. Correlation analyses of the among-group differences in regional CBF-FCS coupling with and without controlling for hypertension and diabetes. (A) The scatter plot of correlation analyses between the *F*-scores of ANOVA with (y-axis) and without (x-axis) controlling for hypertension and diabetes. (B, C, D) The scatter plots showing the correlation analyses between the *t*-scores of the moderate WMH group vs the mild WMH group (B), the *t*-scores of the severe WMH group vs the mild WMH group (C), and the *t*-scores of the severe WMH group vs the moderate WMH group (D), with (y-axis) and without (x-axis) controlling for hypertension and diabetes.

r, Pearson's correlation coefficient. CBF, cerebral blood flow; FCS, functional connectivity strength.

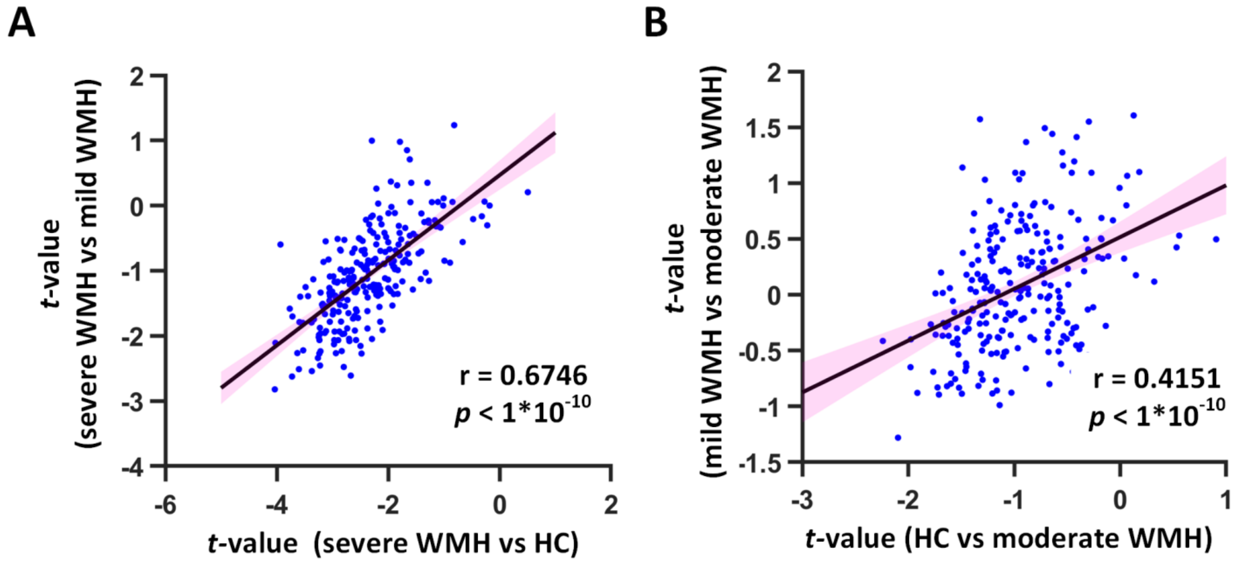

Supplementary Figure 4. The correlation analyses of between-group differences in regional CBF between the HC group vs the moderate/severe WMH group and the mild WMH group vs moderate/severe WMH group. (A) The scatter plot showing the correlation analyses between the *t*-scores of the severe WMH group vs the HC group and the *t*-scores of the severe WMH group and the mild WMH group. (B) The scatter plot showing the correlation analyses between the *t*-scores of the HC group vs the moderate WMH group and the *t*-scores of the mild WMH group and the moderate WMH group. Pearson's correlation coefficient of *t*-scores of *t*-test between groups (with age and sex controlled) was calculated at the regional level. The results indicated that whether compared to the mild WMH group or to the HC group, regional CBF in subjects with higher WMH loads (the moderate WMH group or the severe WMH group) exhibited high consistency on alteration patterns.

r, Pearson's correlation coefficient. CBF, cerebral blood flow.

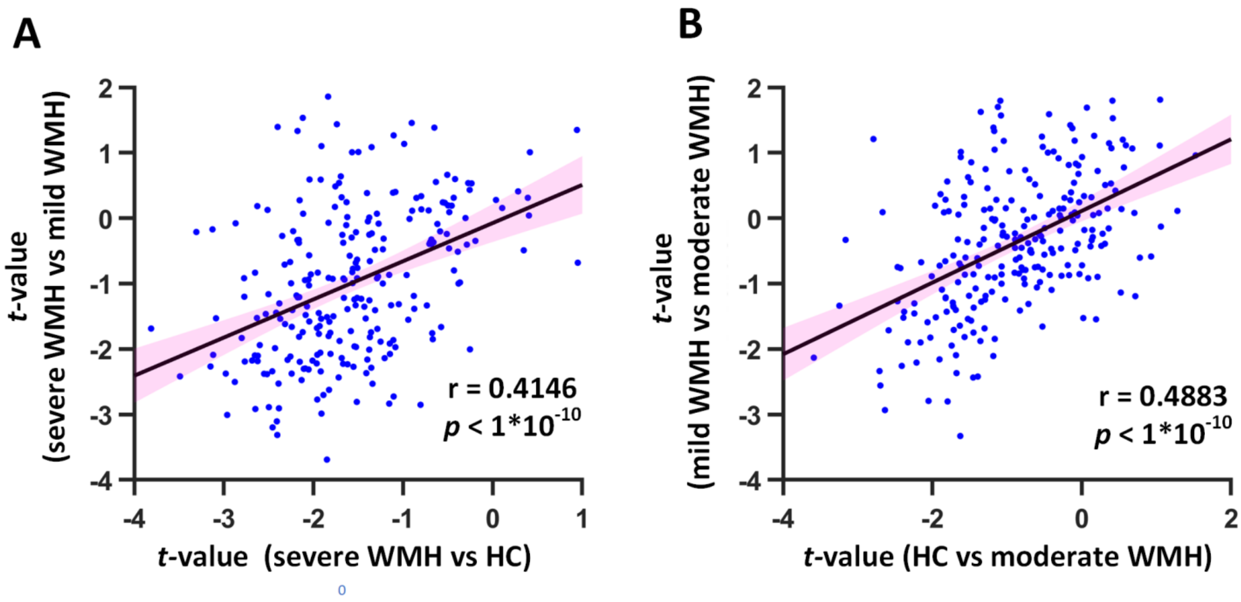

Supplementary Figure 5. The correlation analyses of between-group differences in regional FCS between the HC group vs the moderate/severe WMH group and the mild WMH group vs moderate/severe WMH group. (A) The scatter plot showing the correlation analyses between the *t*-scores of the severe WMH group vs the HC group and the *t*-scores of the severe WMH group and the mild WMH group. (B) The scatter plot showing the correlation analyses between the *t*-scores of the HC group vs the moderate WMH group and the *t*-scores of the mild WMH group and the moderate WMH group. Pearson's correlation coefficient of *t*-scores of *t*-test between groups (with age and sex controlled) was calculated at the regional level. The results indicated that whether compared to the mild WMH group or to the HC group, regional FCS in subjects with higher WMH loads (the moderate WMH group or the severe WMH group) exhibited high consistency on alteration patterns.

r, Pearson's correlation coefficient. FCS, functional connectivity strength.

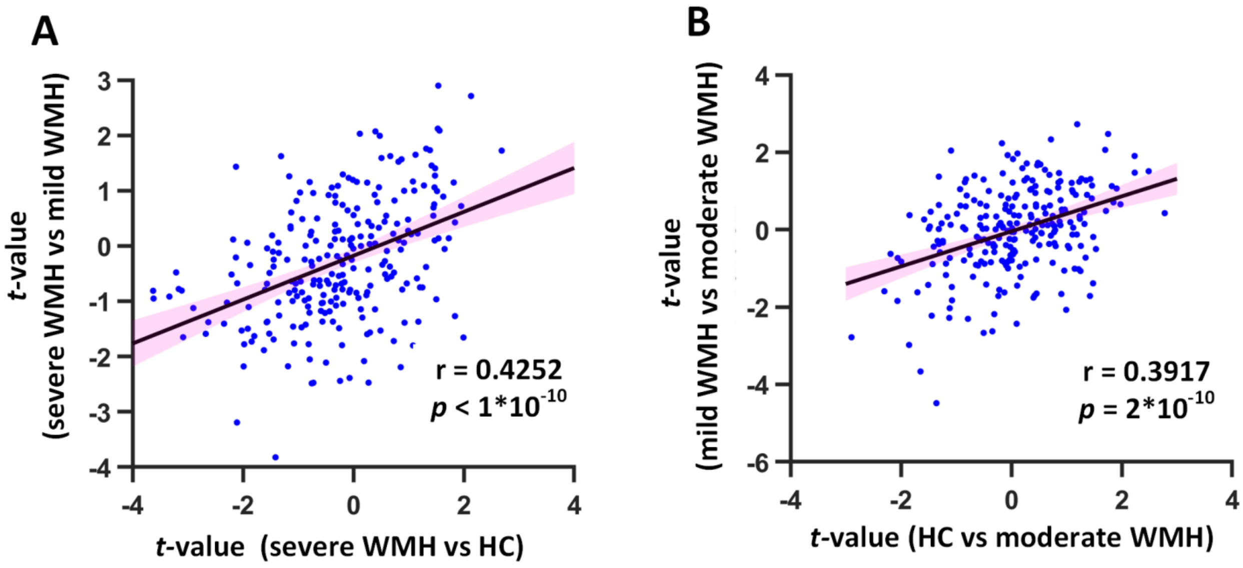

Supplementary Figure 6. The correlation analyses of between-group differences in regional CBF-FCS coupling between the HC group vs the moderate/severe WMH group and the mild WMH group vs moderate/severe WMH group. (A) The scatter plot showing the correlation analyses between the *t*-scores of the severe WMH group vs the HC group and the *t*-scores of the severe WMH group and the mild WMH group. (B) The scatter plot showing the correlation analyses between the *t*-scores of the HC group vs the moderate WMH group and the *t*-scores of the mild WMH group and the moderate WMH group. Pearson's correlation coefficient of *t*-scores of *t*-test between groups (with age and sex controlled) was calculated at the regional level. The results indicated that whether compared to the mild WMH group or to the HC group, regional CBF-FCS coupling in subjects with higher WMH loads (the moderate WMH group or the severe WMH group) exhibited high consistency on alteration patterns.

r, Pearson's correlation coefficient. CBF, cerebral blood flow. FCS, functional connectivity strength.
